# Use of Telephone and Digital Channels to Engage Socioeconomically Disadvantaged Adults in Health Disparities Research Within a Social Service Setting: Cross-Sectional Study

**DOI:** 10.2196/16680

**Published:** 2020-04-01

**Authors:** Kassandra I Alcaraz, Rhyan N Vereen, Donna Burnham

**Affiliations:** 1 Behavioral and Epidemiology Research Group American Cancer Society Atlanta, GA United States; 2 United Way of Greater Atlanta Atlanta, GA United States

**Keywords:** cross-sectional studies, electronic mail, health status disparities, health care disparities, internet, mobile phone, telephone, text messaging, social services

## Abstract

**Background:**

Engaging socioeconomically disadvantaged populations in health research is vital to understanding and, ultimately, eliminating health-related disparities. Digital communication channels are increasingly used to recruit study participants, and recent trends indicate a growing need to partner with the social service sector to improve population health. However, few studies have recruited participants from social service settings using multiple digital channels.

**Objective:**

This study aimed to recruit and survey 3791 adult clients of a social service organization via telephone and digital channels. This paper aimed to describe recruitment outcomes across five channels and compare participant characteristics by recruitment channel type.

**Methods:**

The Cancer Communication Channels in Context Study recruited and surveyed adult clients of 2-1-1, a social service–focused information and referral system, using five channels: telephone, website, text message, web-based live chat, and email. Participants completed surveys administered either by phone (if recruited by phone) or on the web (if recruited from digital channels, ie, website, text message, Web-based live chat, or email). Measures for the current analysis included demographic and health characteristics.

**Results:**

A total of 3293 participants were recruited, with 1907 recruited by phone and 1386 recruited from digital channels. Those recruited by phone had a moderate study eligibility rate (42.23%) and the highest survey completion rate (91.24%) of all channels. Individuals recruited by text message had a high study eligibility rate (94.14%) yet the lowest survey completion rate (74.0%) of all channels. Sample accrual goals were achieved for phone, text message, and website recruitment. Multivariable analyses found differences in participant characteristics by recruitment channel type. Compared with participants recruited by phone, those recruited from digital channels were younger (adjusted odds ratio [aOR] 0.96, 95% CI 0.96-0.97) and more likely to be female (aOR 1.52, 95% CI 1.23-1.88), married (aOR 1.52, 95% CI 1.22-1.89), and other than non-Hispanic black (aOR 1.48, 95% CI 1.22-1.79). Those recruited via phone also were more likely to have more than a high school education (aOR 2.17, 95% CI 1.67-2.82), have a household income ≥US $25,000 a year (aOR 2.02, 95% CI 1.56-2.61), and have children living in the home (aOR 1.26, 95% CI 1.06-1.51). Additionally, participants recruited from digital channels were less likely than those recruited by phone to have public health insurance (aOR 0.75, 95% CI 0.62-0.90) and more likely to report better overall health (aOR 1.52, 95% CI 1.27-1.83 for good-to-excellent health).

**Conclusions:**

Findings indicate the feasibility and utility of recruiting socioeconomically disadvantaged adults from the social service sector using multiple communication channels, including digital channels. As social service–based health research evolves, strategic recruitment using a combination of traditional and digital channels may be warranted to avoid underrepresentation of highly medically vulnerable individuals, which could exacerbate disparities in health.

## Introduction

### Digital Communication and Disparities

Socioeconomically disadvantaged populations bear a disproportionate burden of disease for both infectious and chronic conditions [1-4], yet they remain underrepresented in health research [5,6]. Persistent underrepresentation of populations suffering from disparities in health hinders progress in understanding and eliminating these disparities [7]. Increasingly, digital communication channels such as text message or social media are used to engage individuals in health research [8-11]. Recruitment using digital channels can overcome some of the limitations of traditional recruitment channels (eg, telephone) and has the potential to narrow disparities in health [12,13]. However, evidence-informed strategies are needed to maximally leverage digital technologies for health disparities reduction [13].

Digital communication technologies such as smartphones are increasingly accessible across sociodemographic groups [14-16], including homeless adults [17]. Despite this growing access to digital technology, inequities in technology access continue to be documented among medically vulnerable populations such as low-income individuals [15] and residents of rural communities [16]. Ensuring representation of medically vulnerable populations when recruiting research participants using digital channels, therefore, presents an ongoing challenge.

### Use of Digital Channels in Health Research

Some research suggests socioeconomically disadvantaged populations can be successfully recruited using digital channels, while other findings indicate bias in samples recruited using digital channels. For example, results from a trial of Quit4Baby (a text message–based smoking cessation intervention for pregnant women) demonstrated the feasibility of recruiting high proportions of low-income, unemployed, and publicly insured participants via text message [18]. However, a study comparing characteristics of 12,280 *eCohort* participants recruited on the web to the US population found that participants were more likely to have a college education, less likely to be from racial or ethnic minority groups, and more likely to be in excellent general health [19]. Similarly, a health study employing multichannel recruitment (eg, flyer, email, Facebook, website) found that none of the channels were successful in recruiting individuals of a low socioeconomic status, those from racial or ethnic minority groups, or men [20].

Several trends suggest digital tools will continue to be used in diverse settings to improve population health. First, recent evidence supports the acceptability, feasibility, and efficacy of digital interventions for behavior change [21-27]. Second, also documented is the promise of digital technologies to reduce health care disparities [28]. Third, in 2019, the National Academies of Sciences, Engineering, and Medicine released a report reflecting the growing integration of social care into health care delivery [29]. These trends indicate an emerging demand not only for more health disparities research using digital tools but also for more engagement with the social service sector to enhance health. In recent years, a growing number of researchers have successfully reached and recruited socioeconomically disadvantaged adults for health disparities research through social service organizations [30-37]. However, to our knowledge, scant research to date has employed multiple digital channels to recruit or survey study participants from these types of settings.

### Study Aims

As availability and use of digital channels are increasing, evaluations of web-based recruitment strategies are needed to better understand their effectiveness and potential biases for use in research [38]. Given current trends and needs, understanding how digital channels can be used to engage individuals for health disparities research can enhance research planning. The objective of this study was to recruit and survey a community-based sample of 3791 socioeconomically disadvantaged adults from a social service setting using telephone and digital channels. We also sought to examine recruitment outcomes by channel and participant characteristics by recruitment channel type. We hypothesized that recruitment success would vary across channels and that participant characteristics would vary across the two recruitment channel types (ie, telephone vs digital channels). This paper aimed to present recruitment outcomes for the study and discuss implications for reaching medically vulnerable populations in a social service setting.

## Methods

### Setting

This study was conducted in partnership with 2-1-1, a nationally designated, locally administered information and referral system that connects individuals with resources to meet their basic human and social needs (eg, food, safety). As of 2019, 2-1-1 has been made available to 94.6% of the US population [39] and throughout most of Canada [40]. Where available, individuals can dial 2-1-1 from their phone to request and obtain referrals for services in their local community. Data indicate callers to 2-1-1 are disproportionately low income, uninsured, and unemployed and have high health needs such as for smoking cessation or cancer screening [36,41]. In some communities, 2-1-1 can be reached using digital communication channels such as email or text message.

### Study Overview

This study has reported data from the Cancer Communication Channels in Context (4C) Study, a cross-sectional study that administered a survey to clients of 2-1-1. Survey data from the 4C Study will inform targeted strategies for connecting socioeconomically disadvantaged populations with health- and cancer-related information, programs, and resources. Participants were recruited from United Way 2-1-1 of Greater Atlanta, which was the first 2-1-1 established in the United States. This 2-1-1 Contact Center receives more than 590,000 contacts annually. Individuals can access this Contact Center via telephone, text message, the 2-1-1 website, web-based live chat, email, or a mobile app to request referrals such as a telephone number or website for a community resource. For this study, channels of interest were *telephone* (calling 2-1-1 to request referrals), *website* (searching the self-service 2-1-1 web database), *text message* (texting a referral request to 2-1-1), *web-based live chat* (chatting in real time with 2-1-1 staff via the internet), and *email* (emailing a referral request to 2-1-1). The 4C Study sought to recruit and survey 1895 participants via telephone and 474 via each of the four digital channels (3791 total). These target sample sizes were selected to provide adequate statistical power for primary 4C Study analyses. On the basis of 2-1-1 client volume data, we projected that a 9-month recruitment period would be needed to reach accrual goals.

### Participants

Individuals were eligible for the 4C Study if they were accessing 2-1-1 for referral assistance via 1 of the 5 channels of interest (ie, telephone, website, text message, web-based live chat, email); accessing 2-1-1 from within United Way 2-1-1 of Greater Atlanta’s 13-county primary service area; aged ≥21 years; and able to speak or read English. Exclusion criteria were the following: experiencing an acute crisis (eg, imminent eviction, natural disaster); accessing 2-1-1 on behalf of another person; accessing 2-1-1 in error; or performing a non-English search on the 2-1-1 website.

### Recruitment

Participants were recruited from January to November 2016 by designated 2-1-1 staff who were trained to recruit for the study. Initially, 11 recruiters were designated; 6 recruiters were added in April 2016 to accelerate sample accrual. Individuals were screened for interest and eligibility for the study after receiving standard 2-1-1 service. All individuals searching for referrals on the 2-1-1 website were screened for eligibility; for the other four channels, only those individuals interacting with designated 2-1-1 staff were screened. Screening and recruitment occurred 24 hours per day, 7 days per week.

Recruitment procedures and survey administration mode were based on the communication channel an individual initially utilized to access social services through 2-1-1. Therefore, individuals who contacted 2-1-1 via phone were screened for interest and eligibility during the call. If eligible, informed consent and 4C Study survey administration were conducted immediately after providing the requested 2-1-1 referrals, that is, during the same phone call. Those accessing 2-1-1 using the web-based database received an on-screen notification asking if they were interested in a health survey (yes/no). Those who responded yes received a survey in a new tab where they were screened for eligibility; those who were eligible were directed to a web-based consent page followed by a web-based survey. Individuals who contacted 2-1-1 via chat, text message, or email were sent (via the corresponding channel they used to contact 2-1-1) a statement informing them about a health survey as well as the screener/consent/4C Study survey link. The same survey was used across both survey modes (ie, phone and the web).

Participants were mailed a US $15 gift card incentive after completing the study survey. Participants were also mailed a free resource guide listing free or low-cost health-related cancer prevention services available in their community. Study procedures were approved by the Institutional Review Board at Morehouse School of Medicine.

### Measures

#### Demographic Characteristics

Standard demographic measures included age, sex, educational attainment, marital status, and annual household income. Presence of any children under the age of 18 years living in the home and self-reported race and ethnicity were also assessed. Due to response distribution, race and ethnicity were combined and dichotomized as non-Hispanic black vs other (Hispanic; white; Asian, Native Hawaiian, or Other Pacific Islander; American Indian or Alaskan Native; or other).

#### Health Characteristics

Self-rated health was measured using a standard item: “In general, would you say your health is: excellent, very good, good, fair, or poor?” [42]. To assess health insurance type, respondents were asked to choose which health insurance best describe(s) what they have to help pay their medical bills today. Participants could select more than one response, and responses were recoded into four categories for analysis: uninsured; private; government/public (Medicare, Medicaid, State Children’s Health Insurance Program, Military health care, and/or another government program); or a combination of public and private insurance.

### Statistical Analysis

Analyses aimed to describe recruitment outcomes by recruitment channel (ie, telephone, website, text message, web-based live chat, or email) and participant characteristics by recruitment channel type (ie, telephone or digital channels). First, we examined accrued frequencies and percentages across recruitment channels. For each channel, we computed channel efficiency as the total number of surveys completed divided by the total number of individuals encountered. Second, we compared demographic and health characteristics by recruitment channel type. Means, standard deviations, frequencies, and percentages are presented, along with results of chi-square tests or *t* tests as appropriate. Third, we conducted multivariable binary logistic regression to assess differences in demographic and health characteristics by recruitment channel type while controlling for other characteristics, using phone as the reference group. Adjusted odds ratios and 95% confidence intervals have been presented. All analyses were conducted using SAS software, version 9.4 (SAS Institute Inc).

## Results

### Recruitment

**Figure 1:**
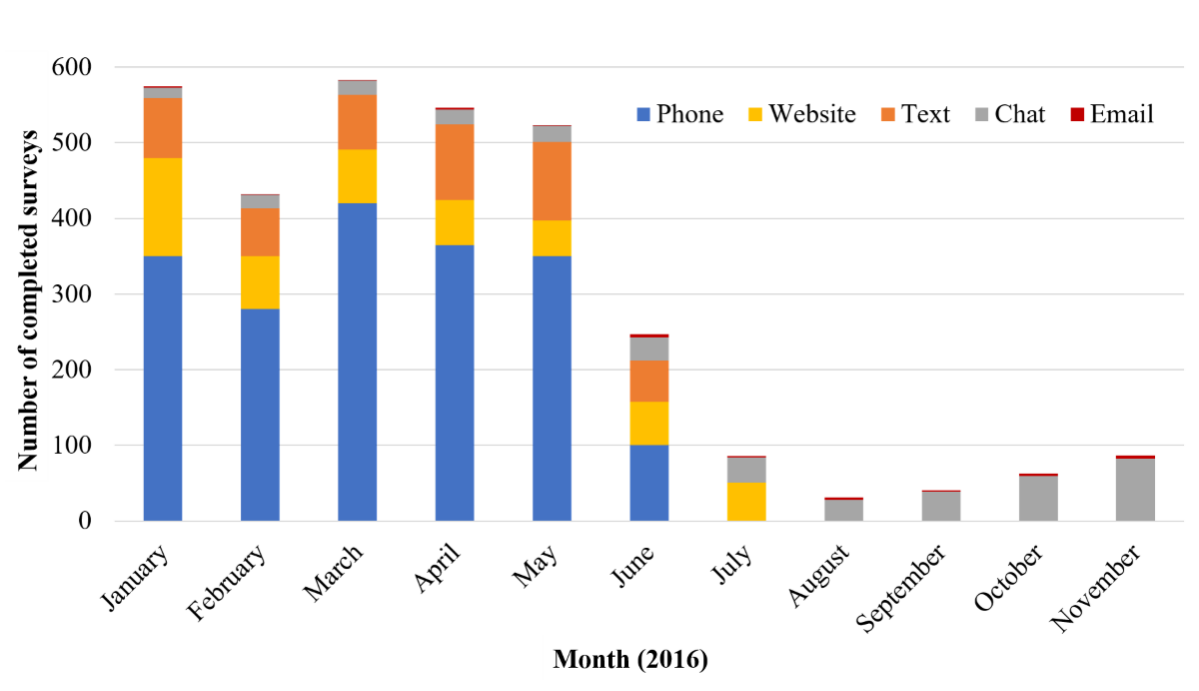
Number of completed Cancer Communication Channels in Context Study surveys per month by recruitment channel, January-November 2016 (accrual goals for phone and text message recruitment were reached in June; accrual goal for website recruitment was reached in July).

Sample accrual goals for the telephone and text message channels were reached in June 2016, and the accrual goal for the website was reached in July 2016. Due to funding constraints, recruitment via web-based chat and email ended in November 2016. Figure 1 depicts the number of participants recruited by month across channels.

**Figure 2:**
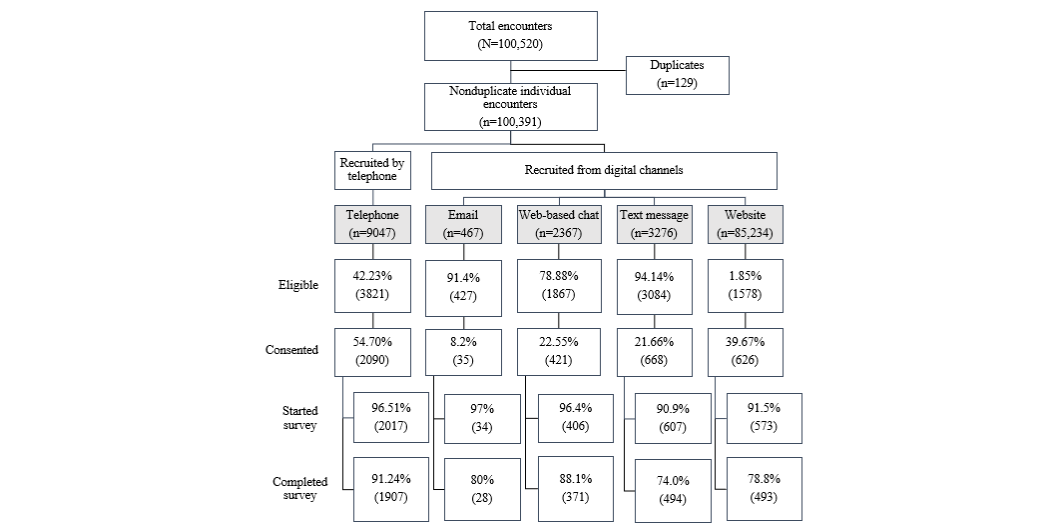
Cancer Communication Channels in Context Study accrual by recruitment channel, January-November 2016.

Figure 2 summarizes sample accrual by recruitment channel. After the exclusion of 129 duplicates, recruiters encountered a total of 100,391 2-1-1 clients. Of these, 10.74% (10,777/100,391) were eligible to participate in the study, 35.63% (3840/10,777) consented to participate, and 94.71% (3637/3840) started the survey. A total of 85.76% (3293/3840) of individuals who consented completed the survey, with 1907 recruited by telephone and 1386 recruited from digital channels.

A wide range of recruitment outcomes was observed across channels (Figure 2). Only 1.85% (1578/85,234) of individuals who were recruited via the website were eligible for the study, compared with 94.14% (3084/3276) and 91.4% (427/467) of individuals who were recruited by text message and email, respectively. Participants who were recruited by phone had the highest survey completion rate (1907/2090, 91.24%), followed by those recruited from web-based live chat (371/421, 88.1%), those recruited by email (28/35, 80%), those recruited from the website (493/626, 78.8%), and those recruited by text message (494/668, 74.0%). Only 28 participants were recruited by email, compared with 371 to 494 for the three other digital channels. Additionally, individuals recruited by email had the lowest consent rate (8.2% vs up to 54.70% for other channels).

The five recruitment channels had a wide range of channel efficiency, with recruitment by phone producing the highest proportion of completed surveys relative to individuals encountered. Specifically, channel efficiency was 21.08% (1907/9047) for phone, 15.67% (371/2367) for web-based live chat, 15.08% (494/3276) for text message, 6.0% (28/467) for email, and 0.58% (493/85,234) for the website. These findings indicate that, to ultimately obtain one completed survey, encounters with 5 individuals on average were required if recruiting by phone; encounters with 7 individuals were required if recruiting from web-based live chat or text message; encounters with 17 individuals were required if recruiting from email; and encounters with 173 individuals were required if recruiting from the website.

### Participant Characteristics

Participant characteristics by recruitment channel type are reported in Table 1. Respondents were predominately female (2662/3293, 80.84%) and non-Hispanic black (2543/3293, 77.22%). Overall, 43.15% (1421/3293) had a high school education or less, and 37.11% (1222/3293) had an annual household income less than US $5000. About half (1566/3293, 47.56%) were never married and 54.02% (1779/3293) had children living in the home. The majority of respondents either had public insurance (1466/3293, 44.52%) or were uninsured (993/3293, 30.15%). About a third of the respondents (996/3293, 30.25%) rated their health as fair or poor.

Demographic characteristics significantly differed by recruitment channel type (Table 1). For example, 16.26% (310/1907) of participants who were recruited by phone had less than a high school education, compared with 8.66% (120/1386) of those who were recruited from digital channels (*P*<.001). Additionally, 41.85% (798/1907) of respondents recruited by phone had a household income less than US $5000, compared with 30.59% (424/1386) of respondents recruited from digital channels (*P*<.001).

Health characteristics also differed by recruitment channel type (Table 1), where 49.66% (947/1907) of respondents recruited by phone had public insurance, compared with 37.45% (519/1386) of respondents recruited from digital channels (*P*<.001). More than a third (706/1907, 37.02%) of respondents recruited by phone rated their health as fair or poor, compared with 20.92% (290/1386) of respondents who were recruited from digital channels (*P*<.001).

**Table 1:** Characteristics of Cancer Communication Channels in Context Study participants by recruitment channel type.

Characteristic		Total (N=3293)^a^	Recruited by telephone (n=1907)^a^	Recruited from digital channels (n=1386)^a,b^	*P* value
Age (years), mean (SD)		42.1 (12.72)	44.8 (13.26)	38.4 (10.87)	<.001
**Sex, n (%)**					<.001
	Female	2662 (80.84)	1479 (77.56)	1183 (85.35)	
	Male	631 (19.16)	428 (22.44)	203 (14.65)	
**Race and ethnicity, n (%)**					<.001
	Non-Hispanic black	2543 (77.22)	1549 (81.23)	994 (71.72)	
	Other	688 (20.89)	345 (18.09)	343 (24.75)	
**Educational attainment** **, n (%)**					<.001
	Less than high school	430 (13.06)	310 (16.26)	120 (8.66)	
	High school graduate or equivalent	991 (30.09)	683 (35.82)	308 (22.22)	
	More than high school	1859 (56.45)	912 (47.82)	947 (68.33)	
**Annual household i** **ncome (US $), n (%)**					<.001
	Less than 5000	1222 (37.11)	798 (41.85)	424 (30.59)	
	5000 to 14,999	874 (26.54)	543 (28.47)	331 (23.88)	
	15,000 to 24,999	546 (16.58)	290 (15.21)	256 (18.47)	
	25,000 or more	500 (15.18)	196 (10.28)	304 (21.93)	
**Marital status, n (%)**					<.001
	Never married	1566 (47.56)	906 (47.51)	660 (47.62)	
	Divorced, widowed, or separated	1071 (32.52)	712 (37.34)	359 (25.90)	
	Married or have a partner	631 (19.16)	284 (14.89)	347 (25.04)	
**Has any children** **in the home, n (%)**					<.001
	No	1499 (45.52)	992 (52.02)	507 (36.58)	
	Yes	1779 (54.02)	914 (47.93)	865 (62.41)	
**Health insurance type, n (%)**					<.001
	Uninsured	993 (30.15)	546 (28.63)	447 (32.25)	
	Public	1466 (44.52)	947 (49.66)	519 (37.45)	
	Private	710 (21.56)	348 (18.25)	362 (26.12)	
	Public and private	86 (2.61)	55 (2.88)	31 (2.24)	
**Self-rated health, n (%)**					<.001
	Poor	233 (7.08)	177 (9.28)	56 (4.04)	
	Fair	763 (23.17)	529 (27.74)	234 (16.88)	
	Good	1078 (32.73)	560 (29.37)	518 (37.37)	
	Very good	750 (22.78)	378 (19.82)	372 (26.84)	
	Excellent	463 (14.06)	260 (13.63)	203 (14.65)	

^a^Column percentages may not total 100% due to missing data.

^b^Digital channels were website, text message, web-based live chat, and email.

### Characteristics Associated With Recruitment Channel Type

Table 2 presents a multivariable logistic regression model comparing demographic and health characteristics of participants who were recruited from digital channels compared with those who were recruited by phone. Respondents who were recruited from digital channels were more likely than respondents recruited by phone to be younger, female, other than non-Hispanic black, have more than a high school education, have higher incomes, be married or have a partner, or have children in the home (all *P*<.05). Additionally, compared with respondents who were recruited by phone, respondents who were recruited from digital channels were less likely to have public health insurance and more likely to report better self-rated health (all *P*<.05).

**Table 2:** Logistic regression model for characteristics associated with recruitment channel type, using phone as the reference category.

Demographics			Recruited from digital channels^a^, aOR^b^ (95% CI)
Age (years)			0.96 (0.96-0.97)^c^
**Sex**			
	Male	1.00 (reference)	
	Female	1.52 (1.23-1.88)^c^	
**Race and ethnicity**			
	Non-Hispanic black	1.00 (reference)	
	Other	1.48 (1.22-1.79)^c^	
**Educational attainment**			
	Less than high school graduate	1.00 (reference)	
	High school graduate or equivalent	1.06 (0.80-1.39)	
	More than high school graduate	2.17 (1.67-2.82)^c^	
**Annual household** **income (US $)**			
	Less than 5000	1.00 (reference)	
	5000 to 14,999	1.21 (0.99-1.48)	
	15,000 to 24,999	1.48 (1.18-1.85)^c^	
	25,000 or more	2.02 (1.56-2.61)^c^	
**Marital status**			
	Never married	1.00 (reference)	
	Divorced, widowed, or separated	0.98 (0.81-1.20)	
	Married or have a partner	1.52 (1.22, 1.89)^c^	
**Has any children** **in the home**			
	No	1.00 (reference)	
	Yes	1.26 (1.06-1.51)^c^	
**Health** **insurance** **type**			
	Uninsured	1.00 (reference)	
	Public	0.75 (0.62-0.90)^c^	
	Private	0.87 (0.69-1.09)	
	Public and private	0.77 (0.46-1.30)	
**Self-rated health**			
	Poor or fair	1.00 (reference)	
	Good, very good, or excellent	1.52 (1.27-1.83)^c^	

^a^Digital channels were website, text message, web-based live chat, and email.

^b^aOR: adjusted odds ratio.

^c^Statistically significant; *P*<.05.

## Discussion

### Principal Findings

Numerous studies have compared the effectiveness of digital channel-based recruitment with traditional recruitment methods, yet few have examined recruitment outcomes across multiple digital channels, particularly in social service settings. To our knowledge, the 4C Study is the first study of social service clients recruited using multiple digital communication channels. The study aimed to recruit 3791 socioeconomically disadvantaged adults across five channels within a social service setting. Recruitment goals were met for 3 of the 5 channels—all except email and web-based live chat, although the latter had moderate recruitment success. The highest channel efficiency was achieved from recruiting by phone. Among the digital channels, recruitment from the website resulted in the largest number of individual encounters and a high number of completed surveys (despite low channel efficiency). Recruitment by text message produced a comparable number of completed surveys despite fewer individuals encountered (demonstrating higher channel efficiency). In contrast, email recruitment resulted in both a low number of individual encounters and a low number of completed surveys.

### Comparison With Previous Work

Findings can enhance the literature on the use of digital channels in diverse populations for research planning, as the appropriateness of a particular recruitment strategy is influenced by technology preferences among the target population [43,44]. Importantly, the number of individual encounters observed by channel in this study reflects the naturalistic use of these channels by individuals accessing United Way of Greater Atlanta’s (UWGA) 2-1-1, the social service setting in which study recruitment occurred. Within UWGA 2-1-1, most requests for referrals occur via phone (ie, calling the 2-1-1 Contact Center). Recruitment for the study was dependent on individuals employing the selected channels to reach or use 2-1-1. For example, recruiters encountered only 467 individuals through the email channel during the entire recruitment period, making this channel less suitable for reaching a large volume of clients quickly. When recruitment for this study was implemented, UWGA 2-1-1 had recently implemented text message as a new communication channel option for clients. In recent years, requests for referrals received via email have declined as options to use other digital channels to request referrals have become more popular among UWGA 2-1-1 clients. Nevertheless, the wide variability in rates of study eligibility, informed consent, and survey completion suggest variability in reach across populations using these channels.

The overall sample recruited reflects the client population served at the recruitment site, which is predominantly female, racial and ethnic minority adults. However, similar to this study, previous research found variation in characteristics of socioeconomically disadvantaged populations across recruitment channel types. A comparison of in-person vs web-based recruitment of adults of low socioeconomic status found that 45% of those recruited in person had annual incomes of <US $10,000 compared with only 16% of those recruited through the web [45]. Thus, even in a targeted recruitment effort, proactively identifying potential bias in a recruitment channel is important for research planning. As noted by Safi et al [45], although different types of recruitment channels may reach socioeconomically disadvantaged participants generally, the channels may differ in the *extent* of disadvantage among participants recruited by each.

In this study of social service clients—a largely socioeconomically disadvantaged group overall—multichannel recruitment resulted in potentially important demographic and health differences between samples from each channel type. The study found that certain channels were more or less likely to recruit participants representative of the local social service client population. The sample recruited from digital channels generally was younger and comprised higher proportions of individuals who were female, married, other than non-Hispanic black, had higher education and income, and had children living in the home compared with the sample recruited by phone. Participants recruited by phone were generally less healthy than those recruited from digital channels and comprised a larger proportion of publicly insured individuals. Similar to these findings, previous research has found that multichannel recruitment is advantageous for recruiting a demographically heterogeneous sample and, in particular, for ensuring representation of underserved populations [44,46-48].

Findings have implications for future health disparities research in social service settings. The findings of this study suggest that future studies may need to recruit across multiple channels (as available in the social service setting) to ensure participants reflect the broader client population. Conversely, for studies requiring targeted recruitment, some channels may provide better access to the target population than others in terms of client volume and/or characteristics. In this study’s setting, findings suggest that targeted recruitment of married individuals or adults with higher educational attainment may be more efficient using digital channels, whereas recruitment by phone may be more efficient for recruiting older adults or individuals with poorer health. Channel type is just one possible strategy to consider for targeted recruitment planning. Other data, such as an individual’s social service needs [49] can be used to profile prospective participant subgroups. Additional research is needed *within* socioeconomically disadvantaged populations and across diverse recruitment venues, such as social service settings, to optimize recruitment outcomes for health disparities research.

Understanding barriers and facilitators to adoption of digital tools across diverse populations can inform research planning. The social service and health care sectors are expected to become more integrated [29] in the immediate future. Digital channels are likely to be used increasingly in both sectors—not only for research recruitment but also for intervention (although more economic research is needed to support, for example, the use of mobile health behavioral interventions [50,51]). One factor that can hinder the impact of digital tools is any channel’s utilization rate in a population, which was observed in this study for email-based recruitment. It is unclear whether the low email engagement rates among clients in the study reflect the low use of email generally or the low use of email for interacting with the 2-1-1 system specifically. Additional research is needed to better understand the factors driving digital technology use in socioeconomically disadvantaged groups including social service clients. It also is unclear whether the reasons for low email channel use are because of preference or access. Inequitable access to technology is another factor than can hinder the reach of digital tools [52]. Some evidence suggests that lack of consistent internet access may present a barrier to certain communication channels among socioeconomically disadvantaged adults [35,53]. Evidence is needed to inform strategies that reduce inequitable access and use of digital tools.

### Limitations

Several potential study limitations must be considered. First, the sample was limited to a single site. In addition, awareness of the availability of the 2-1-1 system may make the 2-1-1 client population different from other socioeconomically disadvantaged adults. Therefore, results may not be generalizable to other populations or settings. However, findings provide some insight into recruitment of socioeconomically disadvantaged populations from the social service sector using multiple communication channels. Second, the cross-sectional nature of the study might not reflect current trends in use of digital channels by UWGA 2-1-1 clients or other social service client populations. As digital technology is ever-evolving, future research is needed to provide evidence on temporal trends in availability and use of digital communication in specific groups and settings. Third, the requirement that individuals who were recruited by phone were required to complete the survey during the call could have biased the sample due to some otherwise eligible individuals not having time to complete the survey immediately. Nevertheless, the demographics of the sample are generally similar to the client population of the recruitment site. Finally, recruitment by channel was dependent on incoming referral requests to UWGA 2-1-1, where client volume per channel varies. However, because recruitment occurred 24 hours per day and 7 days per week, the patterns of encounters observed in the study generally reflect per-channel client volume at UWGA 2-1-1 during the recruitment period.

### Conclusions

Digital communication is increasingly ubiquitous. Concomitant with this trend is the growing availability of digital communication in social services provision [54], offering clients and service providers an array of channels for communicating and accessing or providing services. This study had varying degrees of recruitment success using digital channels to recruit socioeconomically disadvantaged clients of a social service organization over an 11-month period. Recruitment success, in part, reflects patterns of channel use among clients. Accordingly, client volume by channel should be considered in recruitment planning. Recruitment planning also can be informed by understanding the likelihood of a given recruitment channel to engage prospective participants with specific demographic or health characteristics. Overall, findings demonstrated the feasibility of recruiting a sample of socioeconomically disadvantaged adults from a social service setting using digital communication channels, particularly when a channel is well utilized among clients.

Difficulty engaging underserved populations for health research is widely reported in the literature [55]. Despite some channel-specific limitations, the 4C Study recruited and surveyed thousands of socioeconomically disadvantaged adults within a social service setting for a health disparities research study. Recommended strategies for reaching populations underrepresented in research include having direct or derived rapport with potential participants [48] and engaging community organizations or other trusted sources relevant to the population of interest [12,56,57]. Partnering with a social service organization trusted by the study population likely contributed to the study’s overall recruitment success. Ongoing multisector collaboration, coupled with a more nuanced understanding of populations suffering disparities in health, can help overcome persistent recruitment challenges and, ultimately, help eliminate health-related disparities.

## Abbreviations

4C: Cancer Communication Channels in Context
aOR: adjusted odds ratio
UWGA: United Way of Greater Atlanta
